# Supplementary figures and images for: Genome-Wide Identification of Calmodulin-Binding Protein 60 Gene Family and the Function of GhCBP60B in Cotton Growth and Development and Abiotic Stress Response
Source: Int J Mol Sci. 2024 Apr 15;25(8):4349. doi: 10.3390/ijms25084349 (PMC11049924; doi:10.3390/ijms25084349)

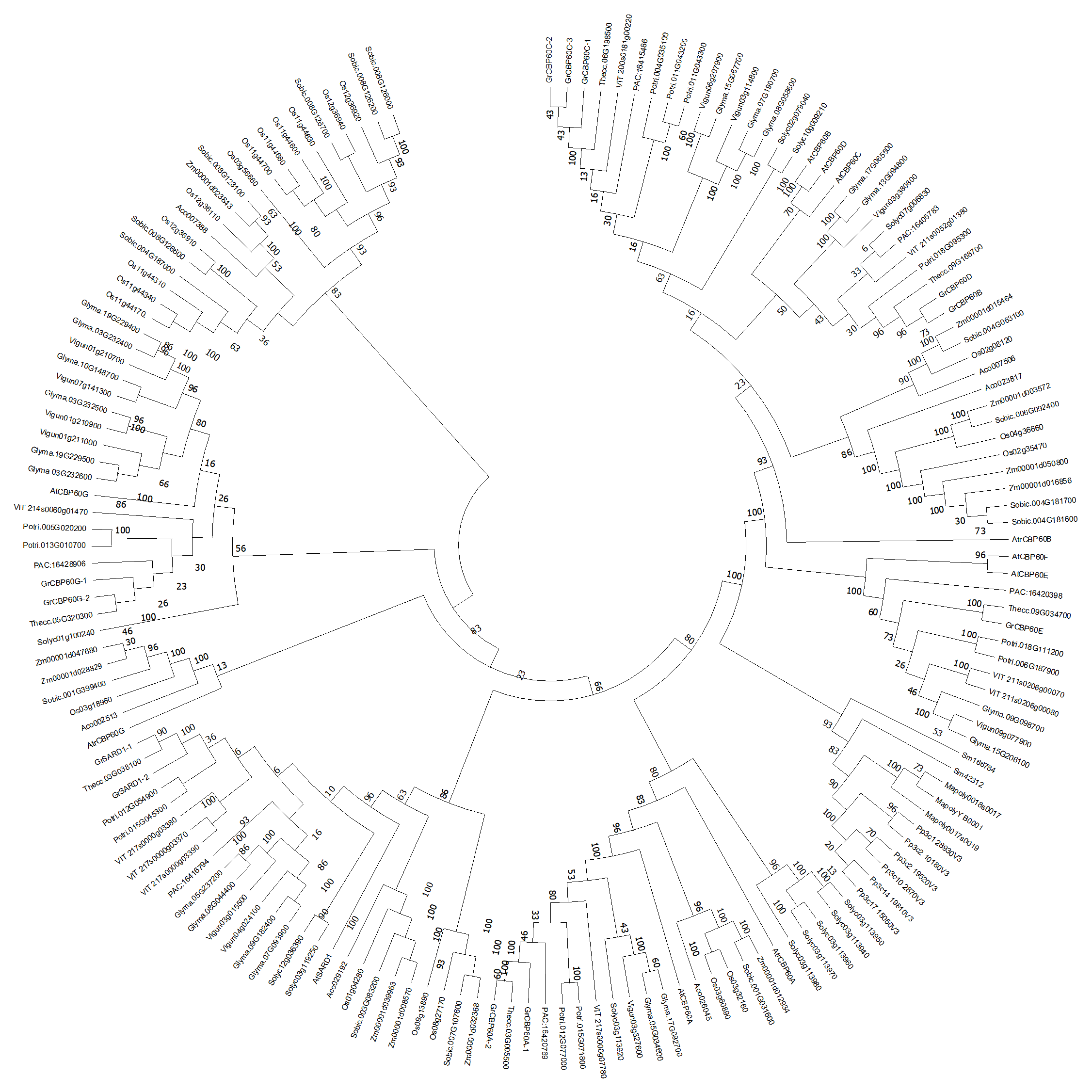

Supplement: Supplementary file 1 [file ijms-25-04349-s001.zip › Figure S1.png]

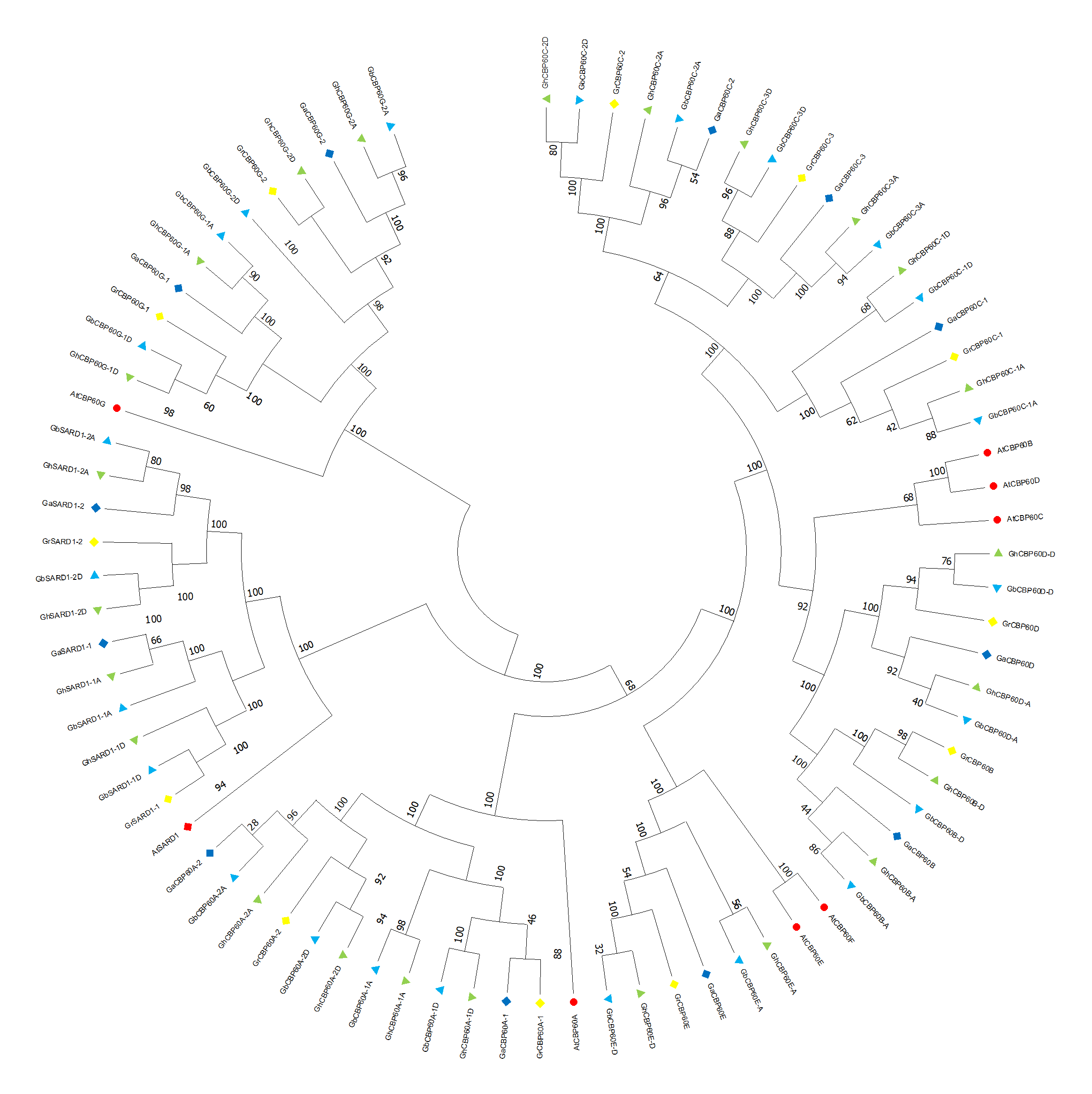

Supplement: Supplementary file 1 [file ijms-25-04349-s001.zip › Figure S2.png]
